# Williamson on conditionals and testimony

**DOI:** 10.1007/s11098-022-01874-7

**Published:** 2022-09-30

**Authors:** Karolina Krzyżanowska, Igor Douven

**Affiliations:** 1 Department of Philosophy & Institute for Logic, Language and Computation, University of Amsterdam, Amsterdam, The Netherlands; 2 IHPST/CNRS, Panthéon–Sorbonne University, Paris, France

**Keywords:** Indicative conditionals, Material conditional, Testimony, Heuristics

## Abstract

In *Suppose and Tell*, Williamson makes a new case for the material conditional account. He tries to explain away apparently countervailing data by arguing that these have been misinterpreted because researchers have overlooked the role of heuristics in the processing of conditionals. Cases involving the receipt of apparently conflicting conditionals play an important dialectical role in Williamson’s book: they are supposed to provide evidence for the material conditional account as well as for the defeasibility of a key procedure underlying our everyday assessments of conditionals. We argue that they can serve neither of these purposes and that Williamson overestimates the reach of heuristics. We specifically challenge Williamson’s assumption that, in the kind of cases centrally at issue in his book, the recipient of conflicting conditionals will typically accept those at face value, even granting Williamson that conditionals can be freely passed among speakers under normal conditions of testimony.

In *Suppose and Tell: The Semantics and Heuristics of Conditionals* (Williamson, 2020),^1^ Timothy Williamson makes a bold attempt to revitalize enthusiasm for the material conditional account (MCA), according to which an indicative conditional has the truth conditions of the corresponding material conditional.^2^ The erstwhile popular account came under a cloud because of apparent conflicts with well-established data about how we use conditionals, most notably, about the conditions under which people are willing to accept and assert a conditional, about the probabilities people assign to conditionals, and about the inferences people are willing to make on the basis of their accepting a conditional.^3^ In Williamson’s new proposal, however, these conflicts are indeed only apparent, and are due to the fact that in using conditionals we rely heavily on heuristics that, although reliable, at times lead us astray.

In Williamson’s view, the primary heuristic for assessing conditionals is the

**Suppositional Procedure (SP)**: To assess a conditional, suppose its antecedent and then assess its consequent; make whatever attitude you take towards the consequent in that hypothetical state your non-hypothetical attitude towards the conditional.

In the literature on conditionals, this is commonly called “the Ramsey Test,” which has served as a vantage point for formal semantics of conditionals (Stalnaker, 1968) and accounts of the probabilities of conditionals (see Edgington, 1995, and Evans and Over, 2004, for more on this). Williamson is the first to interpret it as a fallible heuristic. SP can also be performed in reverse; that is, one can first form an unconditional attitude towards a conditional and then, on its basis, form an attitude towards that conditional’s consequent, given its antecedent. A more general formulation of the suppositional heuristic that, unlike SP, does not give temporal or causal priority to conditional attitudes is the

**Suppositional Rule (SR)**: “Take an attitude unconditionally to ‘If A, C’ just in case you take it conditionally to C on the supposition A” (p. 19).

In other words, one’s attitudes towards a conditional and the corresponding conditional attitudes should be identical.

Williamson adds to this a secondary heuristic:

**Testimony**: Conditionals can be accepted on the basis of other speakers’ testimony, under normal conditions for testimony.

What this means is that if you trust a speaker, then if that speaker reports “If A, C,” you are normally licensed to accept this conditional, and also to pass it on to others. In this regard, conditionals are no different from non-conditional statements.

According to Williamson, the two heuristics can pull in opposite directions: the same conditional may be rendered acceptable or unacceptable, depending on which heuristic the recipient applies. Or more specifically, the Suppositional Rule can be “overridden by weightier epistemic sources, such as authoritative testimony” (p. 93). To support this claim, Williamson deploys a number of variants of a scenario devised by Gibbard (1981) to argue against the view that conditionals have truth conditions. While some theorists followed Gibbard’s path (e.g., Edgington, 1995), contextualists took his scenario to show that the proposition a conditional expresses depends on the speaker’s epistemic state (Kratzer, 2012; Stalnaker, 1984). Williamson’s discussion of Gibbard’s story and its variants is meant to demonstrate that followers of either of these paths are mistaken, because—he argues—they “have implicitly relied too much on the Suppositional Rule, prioritising one fallible heuristic to the exclusion of another, reliance on conditional testimony under normal conditions” (p. 99).

Central to Gibbard’s argument is the principle of conditional non-contradiction (CNC), according to which “If A, C, and if A, not C” is a contradiction unless A is inconsistent. While CNC is intuitively appealing, Gibbard showed that it is possible to construct a scenario in which two conditionals with shared antecedent and contradictory consequents appear to both be true. Williamson bases much of his discussion on the following version of Gibbard’s story, set at a nuclear power plant (p. 89 f):

There has been an accident at a dodgy nuclear power plant. Several warning lights are connected to a single detector beside the nuclear core. When the detector is working and detects overheating in the core, each light is red. When the detector is working and does not detect overheating in the core, each light is green. When the detector is not working, each light is red or green at random, independently of the others. A competent engineer, East, sees only the east light, which is red, and says:

1. If the detector is working, the core is overheating.
  Another competent engineer, West, not in contact with East, sees only the west light, which is green, and says:
2. If the detector is working, the core is not overheating.

As in Gibbard’s original scenario, although both speakers have incomplete information, neither is mistaken about anything relevant. Arguably, East knows (1) and West knows (2), and so both (1) and (2) must be true, which violates CNC.

Following Gibbard, Williamson adds a recipient of the two reports, the Controller, who fully trusts both informants and accepts both reports. She might even repeat both reports out loud, to a colleague or just to herself. Consequently, she infers—correctly, we are told—that the antecedent of the two conditionals is false:

(3) The detector is not working.

This is supposed to show that (1) and (2) can be both accepted and asserted by one speaker in a single context:

What (1) and (2) express in the controller’s mouth depends on her context, not directly on the contexts of the engineers. Anyway, they intended their reports for her context, not just for their own. East expects the controller to accept (1), and West expects her to accept (2), whatever other reports she may receive. Thus (1) and (2) are sometimes acceptable together in a single context, even though they are opposite conditionals, contrary to both the Suppositional Rule and contextualist accounts designed to accommodate CNC. (p. 91)

This scenario and its variants play an important role in Williamson’s defence of the MCA.

In Williamson’s view, Gibbard and other contextualists who propose to treat SR as a constraint on the semantics rather than as a mere cognitive mechanism face the problem that they cannot explain the Controller’s inference to the negation of the antecedent, for to make such an inference, the Controller first needs to accept the two opposing conditionals. And while contextualism may be able to explain why both engineers say something true, it cannot account for the Controller’s acceptance of their testimonies in a single context, that is, the one the Controller is in.

A more general point seemingly favouring the MCA is that while virtually all major semantics of conditionals invalidate CNC, MCA does not. Although that was generally deemed to be more grist for the mill for MCA opponents, Williamson uses Gibbard’s scenario and its variants to try to sell it as a point in support of MCA, the idea being that, in those stories, the best way to make sense of the recipient’s inference to not-A is to assume that the semantics of conditionals is given by MCA. For then “If A, C” is equivalent to not-A-or-C and “If A, not C” to not-A-or-not-C, from the conjunction of which one readily infers not-A.

In our view, however, this is not necessarily the right moral to draw from those stories. To explain our disagreement with Williamson, we start by noting that his argument for MCA is abductive, meaning that it has the form of an inference to the best explanation. Williamson takes Gibbard’s scenario and its variants to yield the data that a theory of conditionals must explain and then argues that MCA is the only theory that can explain those data, in particular, the Controller’s ability to infer (3) from the two seemingly conflicting testimonies, (1) and (2), in a satisfactory manner. It is doubtful, however, whether what Williamson presents as data must really be taken as such.^4^ At a minimum, it is unclear why opponents of the MCA should want to commit to the assumptions underlying Williamson’s analysis of the Gibbardian scenarios. Indeed, we believe that they can make sense of these scenarios without having to take on board any questionable assumptions. To do so, they should reason abductively, just as Williamson does, but then further insist that the recipient of the seemingly conflicting conditionals in the Gibbardian scenarios is also most plausibly thought of as reasoning abductively in order to figure out what to infer from those conditionals.^5^

Naturally, we have no objections to Williamson’s Testimony heuristic: we do sometimes accept conditionals on the basis of other people’s testimony. But Williamson’s take on what exactly we accept when we accept a conditional on the basis of testimony, and in particular his assumption that, normally, we accept conditionals verbatim and that “there is no general need to reword them to preserve their content” (p. 89), are more doubtful.

Because conditionals may be transferred by means of memory or testimony, it may be possible to find contexts in which there is a discrepancy between what SR and Testimony render acceptable, and we may decide to rely on the testimony of others instead of on our own reasoning. In other words, the Testimony heuristic may overrule SR. This claim appears uncontroversial, at first blush at least: what we may come to believe as a result of our own investigations may differ from what others tell us, and sometimes we may have more confidence in the expertise of others than in the results of our own investigations. If we then admit that we may have made mistakes in ascertaining the facts or in drawing inferences, we may allow someone else’s testimony to overrule our own convictions. There is no reason to assume that conditionals would differ from categorical statements in this respect. However, this is not the kind of discrepancy that Williamson has in mind. For Williamson, the two heuristics may pull in different directions because a material conditional may be highly probable while the corresponding conditional probability—the outcome of SP—is low, and so it is possible for an agent to accept a conditional, particularly when its source is a reliable testimony or memory, without accepting its consequent on the supposition of the antecedent. If we do not accept the material interpretation of the conditional, however, but take the testimony of the conditional to convey, for instance, that C is highly probable given A (as suggested, for instance, by Edgington (1995); see also Oaksford and Chater (2007), and Collins et al. (2020), for empirical support for this view), SR and Testimony will pull in opposite directions only in a situation of genuine disagreement. For instance, if we already believe not-C to a high degree conditional on A, it is reasonable to suppose that we will either disregard the source of the opposing testimony as incompetent or untrustworthy or accommodate the new information by revising our own (conditional) degrees of belief.

Still, grant, for the sake of argument, that it is indeed possible for the two heuristics to pull in different directions in Williamson’s sense. Why would Testimony then override SR when the information we receive from others conflicts with other testimonies, and possibly also with our own judgment? This is precisely what is supposed to be happening in Williamson’s variant of Gibbard’s story: the Controller does not know what kind of evidence the two engineers based their assertions on, but she trusts them completely and so “simply accepts both reports,” which, according to Williamson, puts her in a position to “reasonably and correctly [conclude] (3) from (1) and (2)” (p. 91). Williamson presents the Controller’s behaviour as a simple fact of the matter, a piece of evidence that a theory of conditionals must explain, yet, unless we already believe that the Testimony heuristic results in people’s accepting material conditionals, we may question the plausibility of Williamson’s take on how the Controller would reason.

That the Controller simply infers the negation of the antecedent from two conflicting conditional testimonies is not a piece of evidence; it is an assumption of Williamson’s argument. Moreover, Williamson maintains that the Controller needs to be able to accept both conditionals “together in a single context” (p. 91) which, it seems, is a reasonable thing to do only under the assumption of MCA, considering that on other known accounts one cannot simultaneously believe “If A, C” and “If A, not C” without inconsistency. In the remainder of the paper, we will focus on the main assumption of Williamson’s argument, namely, that when we receive “If A, C” and “If A, not C” from two reliable sources, we simply infer not-A. We will argue that neither the descriptive nor the normative part of this assumption is defensible. This will also involve arguing that, even if we grant Williamson that people do make such inferences, it does not mean that people need to be able to accept both conditionals at the same time, in a single context, and without any rewording.

Consider the following scenario: It is the opening stage of a chess game, with Magnus Carlsen (white) and Hikaru Nakamura (black). Carlsen’s second observes that Carlsen can make a move (say, Bishop g2 to h3) to create a situation which, in current grandmaster practice, has always led to a significant advantage for white. Nakamura’s second also sees that Carlsen is in a position to play Bg2–h3. But he knows that Nakamura has recently been studying the resulting position and has found a refutation, which leads to a position that is much better for him instead. Now the room is cleared. Carlsen’s second reports: “If Carlsen played Bg2–h3, he is in the best position for winning the game.” Nakamura’s second tells us: “If Carlsen played Bg2–h3, Nakamura is in the best position for winning the game.” We trust both speakers. Should we now conclude that Carlsen did not play Bg2–h3? If we accept both reported conditionals, then according to MCA, that is what we should do indeed. But we may assume that Carlsen is unaware of Nakamura’s recent discovery. So, if he saw that he could play Bg2–h3, he may well have made the move. On the other hand, perhaps he did not see the possibility, or he did see it but he has a spy in Nakamura’s camp and therefore knows that the move would backfire, or he saw the possibility but saw another possibility as well, which he thought was even more likely to result in a win. In light of these considerations, it would seem unwise to infer anything with respect to Carlsen’s playing or not playing Bg2–h3 from the two conditionals reported to us. The point is not that these specific considerations will go through our mind when pondering what to make of the apparently conflicting conditionals. But we may reckon with the fact that neither Carlsen’s nor Nakamura’s second has possession of *all* the data relevant to the issue of who is in the best position to win if Carlsen played Bg2–h3, which may be enough to block us from making any inference.

In this example, it is best to think of our accepting the two conditionals as data points of which we are to make sense as best we can. We may be thought to reason abductively. That in the end we do not feel licensed to infer anything from the data concerning either player’s chances of winning, supposing that Carlsen played Bg2–h3, is inessential; that is what most accounts of abduction *recommend* in cases in which there is no clearly best explanation of the available evidence (e.g., Lipton, 1998; Bird, 2000). The important point to notice is that, for all Williamson shows, we proceed in the same way in his variant of the Gibbard scenario in which the Controller is said to be a bureaucrat who knows nothing about the design of the reactor’s warning system. When she receives the reports from East and West, (1) and (2), she may interpret them as the results of, respectively, East’s and West’s own applications of SR. Her reasoning might then go as follows: The supposition that the detector is working led East to the conclusion that the core is overheating, while West, on the same supposition, concluded that it is not. Both engineers are competent and trustworthy, so East’s conclusion must be justified given East’s evidence, and West’s conclusion must be justified given West’s evidence. Because these conclusions contradict each other, they must be based on different evidence. The engineers’ evidence might be simply incomplete, but it might also be conflicting. Because it is already mentioned in the shared antecedent of (1) and (2), the faulty detector is a salient candidate for the source of the conflicting evidence, and so the Controller can conclude that the detector is not working, as Williamson demands. However, given that, as we are told, the Controller has no insight into the evidential basis for East’s and West’s testimonies, she may, and possibly ought to, think of other options, too. Perhaps the detector is actually working, but the signals East and West received from it have been corrupted. Or maybe there is a virus in the software the engineers are using. Furthermore, the Controller might, and perhaps ought to, consider a possibility that only one of the two engineers has been affected by corrupted evidence. While the Controller’s trust in her engineers’ competence might remain unshaken, it is perfectly rational for her to doubt their evidence, given that she is in a clear sense better informed than either of the engineers by having *both* reports. In the end, she will do what makes most sense in light of these considerations, and given all the data she has, most notably, East and West’s testimonies. In Williamson’s variant of the Gibbard scenario, it seems reasonable indeed to infer to what in light of the foregoing considerations appears to be the best explanation of the evidence, viz., that the detector is not working.

To hold that this abductive approach—inferring what makes best sense of the relevant data, including the conditional testimonies, if any inference appears warranted at all—applies generally to Gibbardian scenarios has an important advantage in that it allows us to do without a problematic assumption underlying Williamson’s analysis. We previously saw that, commenting on one of his Gibbardian stories, Williamson (2020, p. 91) says that each of the informants expects the addressee to accept their report, “whatever other reports she may receive.” But consider a simple case, not involving conditionals, in which you tell a friend that A. Are you expecting your friend to accept A no matter how many other friends of hers might simultaneously be telling her that not-A? Or what if someone clearly more expert on the issue of A-or-not-A is telling her that not-A? Or again, if the two authors of the present paper disagree on the matter of A, and one of us tells a common friend that A while the other tells her that not-A, and we have strong reason to believe that our friend trusts us equally, then surely the two of us can only be expecting our friend to accept what she is being told by us if we assume that she is happy to accept contradictory statements (or that she fails to spot the contradiction). We should not expect a different type of response if the reports involve opposing conditionals. If you receive only the report that if A, C, you might accept it without any hesitation. But if at the same time you receive the report that if A, not C, then, presumably, that will give you pause, just as simultaneously receiving A and not-A will give you pause.

To deny that in the kind of situations at play in Gibbardian stories we should expect the hearer to accept the conditionals relayed to her is not necessarily to deny Williamson’s claim that, *under normal circumstances*, conditionals can be freely passed from one speaker to another. After all, the situations in those stories are *not* normal. It is *not* normal to receive, at the same time, conflicting messages. Naturally, it can happen that we receive such messages at the same time, but such situations are special and require special attention.

In fact, recent work in the psychology of reasoning, especially on the role heuristics play in reasoning, makes it natural to think that these situations are likely to *get* special attention. Thanks to this work, dual-processing theories have come to dominate theorizing about how people reason. Such theories contrast heuristic thinking, which is fast and effortless, with analytic thinking, which is slower and requires effort (see, e.g., Evans, 2003; Stanovich, 1999). The currently most widely endorsed dual-processing theories are of a default–interventionist type, according to which heuristic processes yield a default response but then various types of events can trigger an analytic intervention (Evans 2006; Kahneman and Frederick, 2002). At the moment, we are far from having a complete catalogue of what can cause analytical thinking to kick in. But there is strong evidence that processing fluency—the ease with which a person processes incoming information—is a good predictor of whether an analytic intervention will take place (Thompson et al., 2011). Given that, at a minimum, the receipt of conflicting information calls for some kind of conflict resolution, it will impede processing and thus is likely to activate analytical thinking, overriding the default reliance on heuristics, which in the kind of case at issue is possibly captured by Williamson’s Testimony. On our proposal, the analytical thinking will lead someone who receives both “If A, C” and “If A, not C” from trusted sources to treat these conditionals as data points of which she must make some kind of sense, which—we further propose—she will try to do by figuring out what would best explain this data, also taking relevant background knowledge into account. It is indeed extremely implausible that when the primary heuristic, SP, which requires us to employ our own reasoning to assess a conditional, leads us to suspicious conclusions, triggering thereby our slow, analytic thinking processes, we will override the outcome of our own reasoning by applying not just another heuristic, but also one that makes us blindly accept what other people say.

We have argued that while people do accept conditionals on the basis of testimony, we have no good reason to assume that they would do that when the testimonies conflict, or even only seem to conflict. From our perspective, the Controller’s accepting the two testimonies is not a data point that needs to be accounted for but is rather a contentious assumption of Williamson’s argument. We have also argued that inferring not-A, given that we receive “If A, C” and “If A, not C” from two reliable sources, is not always justified, and thus it can neither be treated as a fact about human reasoning, nor as a norm that humans should aspire to in their reasoning. We should emphasize, however, that we are not claiming that such an inference would *never* be justified. For instance, if our Controller is well informed about the detector’s signalling system, she will immediately realize, upon receiving (1) from East and (2) from West, that the east light is red and the west light is green, which unambiguously signals a fault in the detector. Note, though, that in this well-specified context (1) and (2) are equivalent to categorical statements involving indexicals, viz., “The light I see is red” and “The light I see is green,” respectively. Thus, the fact that the Controller can reasonably infer that the detector is not working on the basis of these two testimonies does not provide evidence for the Controller’s accepting the two conditionals verbatim.

To elaborate, in this version of the scenario, even on the assumption that “red” means “not green,” the two engineers do not contradict each other any more than “I was born in Paris” and “I was not born in Paris” contradict each other when uttered by two different speakers. All the Controller needs to do to make her inference is to accept the two categorical statements that are entailed by the conditionals after rephrasing them just enough to fix the referents of the indexical “I,” that is, she can infer that the detector is not working upon accepting “The east light is red” and “The west light is green.” In Williamson’s variant of the story, however, the Controller is not aware of how the signalling system in the nuclear plant is constructed. The engineers’ testimonies are all the data she can rely on.

A final point to stress in relation to our reply to Williamson is that, while we are proposing that the Controller can be thought of as treating the conditionals she receives as data points and not premises of a deductive argument that need to be accepted verbatim, we are not saying that she is not accepting any propositions in that process. We are only saying that the Controller need not accept (1) and (2) in a single context, without doing any rewording to account for context-sensitivity, as Williamson demands. The contextualist can plausibly argue that the Controller accepts the propositions expressed by East’s and West’s utterances of (1) and (2), respectively, *after enriching them with all the contextually provided information*. She may rephrase them, for instance, along the following lines:

(4) Given East’s evidence, if the detector is working, the core is overheating.
(5) Given West’s evidence, if the detector is working, the core is not overheating.

The adjustments the Controller makes in this case are analogous to the adjustments she would make when accepting “The light I see is red” and “The light I see is green.” In both cases, the apparent conflict between the testimonies vanishes.^6^

Williamson would disagree. He insists that whenever we find ourselves in the Controller’s position, we can and often do accept “If A, C” and “If A, not C” as they are, without making any such adjustments, because “for the purposes of communication, conditionals are nothing special”: they are used to express things about the world (p. 89). We have two comments on this. First, we do not claim that conditionals always require the above kind of rephrasing. In normal circumstances of communication, no rephrasing may be called for; for instance, it is often unnecessary to replace indexicals such as “here,” or “now,” or even personal pronouns like “he,” in everyday contexts in which all interlocutors are aware that they are talking about, say, London, the period in British history after Brexit, and Boris Johnson. Importantly, however, the circumstances of Gibbardian stories are, as we have argued above, anything but normal. Second, the need to rephrase or resolve context-dependency does not necessarily make conditionals as special as Williamson seems to suggest, given that, in natural language, semantically underdetermined expressions (i.e., expressions whose surface structure does not provide sufficient information to determine their meaning) are ubiquitous.^7^

To sum up, we can make perfectly good sense of the data from Gibbardian scenarios without committing to MCA, and we can do so without having to assume that Testimony, which is fine in itself, applies in those scenarios. Indeed, Testimony does *not* apply in Gibbardian scenarios because they do not present us with normal conditions of testimony. And if the conditionals received in those cases cannot be expected to be accepted verbatim, then nothing follows regarding SR. Most likely, someone receiving such conditionals will process them analytically, drawing on background knowledge to figure out the relevant conditional probabilities. As we have shown, in some contexts, such a person might even be able to infer the negation of the antecedent as the best explanation of the two speakers’ assertions without accepting the opposing conditionals (or their contextually enriched paraphrases). We conclude that Gibbardian scenarios can play neither of the dialectical roles assigned to them in Williamson’s book.^8^

## Footnotes

1 In the following, all unadorned page references are to this book.

2 From here on, we refer to indicative conditionals simply as “conditionals.”

3 For an overview of early data on these issues, see Evans and Over (2004).

4 In fact, Williamson appears to endorse a “large gap between theory and observation” as a purported feature of modern, sophisticated scientific methodology (p. 116). For insightful critiques of, among other things, Williamson’s fast-and-loose approach to data, see Berto (2022) and Rothschild (2022).

5 As an anonymous referee remarked, while Williamson will not object to our reliance on abduction in this paper, others might well do so. In particular, Bayesians tend to reject abduction as a rational mode of inference. We believe this to be a mistake, however, for reasons canvassed in Douven (2022).

6 The context-sensitivity exhibited by conditionals may be thought to be akin to that of indexicals, as suggested by Stalnaker in his comments on Gibbard’s stand-off (Stalnaker 1984, pp. 109–111). Alternatively, it could be construed in terms of unarticulated constituents as discussed by Recanati (2003).

7 While there has been a heated debate concerning context-sensitivity in language, the opposing parties tend to disagree on the analysis of sentences exhibiting context-sensitivity, not on the existence of the phenomenon itself. See, for instance, Carston (2002), Recanati (2004), and Borg (2004), for different approaches to the subject.

8 In the early stage of this research, Karolina Krzyżanowska was supported by “The Logic of Conceivability (LoC)” Project funded by the European Research Council (ERC CoG), Grant Number 681404.

## References

[CR1] Adams EW (1975). The logic of conditionals.

[CR2] Berto, F. (2022). Williamson on indicatives and suppositional heuristics. *Synthese,**200,* 8. 10.1007/s11229-022-03518-z PMC8853152 35194257

[CR3] Borg E (2004). Minimal semantics.

[CR4] Carston R (2002). Thoughts and utterances: The pragmatics of explicit communication.

[CR5] Collins PJ, Krzyżanowska KH, Hartmann S, Wheeler G, Hahn U (2020). Conditionals and testimony. Cognitive psychology.

[CR6] Douven I (2022). The art of abduction.

[CR7] Edgington D (1995). On conditionals. Mind.

[CR8] Evans JSB (2003). In two minds: Dual process accounts of reasoning. Trends in cognitive sciences.

[CR9] Evans JSB (2006). The heuristic-analytic theory of reasoning: Extension and evaluation. Psychonomic bulletin and review.

[CR10] Evans Jonathan St. B. T., Over David E. (2004). If.

[CR11] Gibbard, A. (1981). Two recent theories of conditionals. In W. L. Harper, R. Stalnaker, & G. Pearce (Eds.), *Ifs* (pp. 211–247). Dordrecht: Reidel.

[CR12] Kahneman D, Frederick S, Gilovich T, Griffin D, Kahneman D (2002). Representativeness revisited: Attribute substitution in intuitive judgement. Heuristics and biases: The psychology of intuitive judgement.

[CR13] Kratzer A (2012). Modals and conditionals.

[CR14] Oaksford M, Chater N (2007). Bayesian rationality: The probabilistic approach to human reasoning.

[CR15] Recanati F (2003). Literal meaning.

[CR16] Rothschild D (2022). Living in a material world: A critical notice of suppose and tell: The semantics and heuristics of conditionals by Timothy Williamson. Mind.

[CR17] Stalnaker RC, Rescher N (1968). A theory of conditionals. Studies in logical theory.

[CR18] Stalnaker R (1984). Inquiry.

[CR19] Stanovich KE (1999). Who is rational? Studies of individual differences in reasoning.

[CR20] Thompson VA, Prowse Turner JA, Pennycook G (2011). Intuition, reason, and metacognition. Cognitive psychology.

[CR21] Williamson T (2020). Suppose and tell: The semantics and heuristics of conditionals.

